# I Wasn’t at War With the Noise: How Mindfulness Based Cognitive Therapy Changes Patients’ Experiences of Tinnitus

**DOI:** 10.3389/fpsyg.2020.00483

**Published:** 2020-04-17

**Authors:** Elizabeth Marks, Paula Smith, Laurence McKenna

**Affiliations:** ^1^Department of Psychology, University of Bath, Bath, United Kingdom; ^2^Department of Psychology, Adult Audiology, Royal National Throat Nose and Ear Hospital, London, United Kingdom

**Keywords:** tinnitus, mindfulness, mindfulness based cognitive therapy, MBCT, IPA

## Abstract

**Objectives:**

Intrusive tinnitus is a challenging, life-changing experience for which traditional medical treatment does not yet have a cure. However, Mindfulness Based Cognitive Therapy for tinnitus (MBCT-t) is effective in reducing tinnitus-related distress, disability and intrusiveness. It is a priority to understand patients’ experience of MBCT-t and active processes which they regarded as underpinning the changes they experienced. Semi-structured interviews were conducted 6 months after participants had completed MBCT as part of a randomized controlled trial (RCT), with a focus on exploring their experiences of the course, what they felt had changed and how they felt such changes had occurred.

**Methods:**

Nine participants took part and Interpretative Phenomenological Analysis (IPA) was used to analyze the interview transcripts.

**Results:**

Four overarching themes emerged: (1) Relating to Tinnitus in a New Way, (2) Holistic Benefits, (3) Connection, Kindness and Compassion, and (4) Factors Supporting Engagement and Change.

**Conclusion:**

All participants reported benefits from MBCT-t, based on a radically new relationship with tinnitus. It was no longer characterized by “fighting it” and was instead based on “allowing” tinnitus to be present. Changes were supported by the development of open, stable, present-moment awareness and attitudes of equanimity, kindness, and compassion. Practices encouraging focus on sound (including tinnitus) were challenging, but essential to learning this new way of being with tinnitus. MBCT-t had a huge range of benefits including reduced distress and enhanced wellbeing. The group nature of MBCT-t was an integral part of the therapeutic process. A number of clinical and research implications are discussed.

## Introduction

Tinnitus is the internal sensation of sound that has no external cause. It is extremely common, with prevalence at 30% (McCormack et al., 2016), and with severe tinnitus reported by 1–2% of the population, it represents a significant public health problem, with millions seeking treatment annually across the globe. Comorbid anxiety, depression, stress, insomnia, poor concentration, and functional disability are commonplace (Baguley et al., 2013). Conventional biomedical approaches fail to “silence” tinnitus and the strongest evidence base is for psychological intervention based on cognitive behavioral therapy (CBT) (Martinez-Devesa et al., 2010; Fuller et al., 2020; and more recently, mindfulness interventions (Rademaker et al., 2019).

Recent clinical standards and guidelines indicate psychological therapies for tinnitus (e.g., Cima et al., 2019). However, patient experiences are not yet in line with recommendations, and a recent review found that the majority of tinnitus patients do not receive sufficient therapeutic assistance for tinnitus (McFerran et al., 2019). Within the UK, only 11% of patients receive CBT and 9% mindfulness meditation, with the majority receiving written information (67%), sound therapy (35%), listening strategies (29%), or relaxation (23%) (McFerran et al., 2018). Thus although guidelines recommend psychological treatments for tinnitus, the standard care available for patients is far more likely to involve strategies based on acoustic approaches and relaxation.

Mindfulness Based Programmes (MBPs) systematically train participants in mindfulness. The best known structured training programme is Mindfulness Based Stress Reduction (MBSR) with decades of evidence in the treatment of chronic illness (Kabat-Zinn, 1990). Mindfulness Based Cognitive Therapy (MBCT) combines this with CBT and was originally developed to treat depressive disorder and relapse (Kuyken et al., 2016). MBCT and MBSR have small to moderate effects on psychological wellbeing in chronic medical conditions (Bohlmeijer et al., 2010).

Mindfulness has been defined as “a way of being in a wise and purposeful relationship with one’s experience… cultivated by systematically exercising one’s capacity for paying attention, on purpose, in the present moment and non-judgmentally” (Mindful Nation UK, 2015). Kabat-Zinn (1990) highlights seven attitudinal foundations for mindfulness (acceptance, non-judging, patience, letting-go, trust, beginner’s mind, and non-striving). Other definitions focus on self-regulation, requiring *sustained attention* (to recognize what arises in each moment), *attentional switching* (flexibility) and *inhibition of secondary elaborative processing* (noticing experience without thinking about it). Integrating several models, Kuyken and Feldman (2019, p. 14) offer a five-faceted definition of mindfulness as:

1. A *state* of being present, a *process* of unfolding moment-by-moment experience *and a faculty* that can be cultivated and applied.
2. Based upon intentionality (about the placement of attention and awareness),
3. Imbued with attitudinal qualities (curiosity, patience, friendliness, care, trust and equanimity).
4. Something that requires effort to cultivate.
5. Intrinsically ethical (i.e., deployed to reduce suffering and enhance wellbeing).

Cultivating mindfulness is proposed to benefit health and wellbeing through various processes. Crane et al. (2017) suggest that it teaches *discernment*: People learn to recognize automatic behaviors and related distress arising from unhelpful habits of mind and choose to respond in more skilful ways, with less reactivity. Supported by empirical study, outcomes in MBPs have been shown to be mediated by reduced cognitive and emotional reactivity, repetitive negative thinking and increased self-compassion and psychological flexibility (e.g., Alsubaie et al., 2017). Probably, universal and specific features of MBCT/MBSR will be differentially relevant across conditions, with acceptance and exposure important in physical conditions (Carlson, 2012) characterized by avoidance, selective attention and catastrophic thinking (e.g., tinnitus; McKenna et al., 2014). Thus processes of change must be examined in specific groups to refine particular applications of MBPs.

Tinnitus distress is maintained by repetitive, catastrophic and negative thinking leading to unhelpful fear-based strategies of suppression, avoidance and distraction (McKenna et al., 2014; Marks et al., 2019). Unhelpful attentional processes include purposeful and automatic selective attention *toward* the feared tinnitus, and difficulties with sustaining attention elsewhere or switching attention away from tinnitus (as attention is “captured” by tinnitus). Unfortunately, early advice given to tinnitus patients often causes iatrogenic distress, as they are told to “just learn live with it, because there is no cure.” This results in feeling both hopeless and responsible for getting rid of or suppressing the noise, which fuel the unhelpful strategies described above (Marks et al., 2019). Standard treatments (with a poor evidence base) can likewise promote unhelpful behaviors by encouraging tinnitus avoidance (e.g., masking, distraction), without targeting underlying cognitive behavioral processes. In contrast, CBT for tinnitus focuses on changing the content of cognitions about tinnitus, reducing stress and increasing exposure to tinnitus.

Mindfulness is a unique approach to tinnitus because it directly targets key processes of sustained and flexible attention. Based on standard MBCT (Segal et al., 2012), MBCT for tinnitus (MBCT-t) includes adaptations focusing on sound and the cognitive model of tinnitus (McKenna et al., 2017). Participants practice developing mindful awareness toward all experience, including tinnitus and practice approaching tinnitus rather than avoiding it. Unlike CBT, the relationship to negative thoughts is changed, rather than the content of them. Participants practice using sustained and flexible attention, choosing to pay attention in a way that is based on choice, receptivity and non-judgment, rather than fear. One might be concerned that paying more attention toward tinnitus would exacerbate distress. However, theoretically, it is the distressing cognitions and stress arousal that drive distress, and narrow attention onto tinnitus. Since mindfulness practice redeploys attention by widening the attentional “spotlight” to include other events and experiences as well as tinnitus, it should result in less distress and awareness of more stimuli than just tinnitus. Any distortion in the perception of tinnitus as a consequence of selective attention and resistance is reduced.

MBCT-t and CBT for tinnitus overlap, sharing an underpinning theory that cognitive-behavioral responses to tinnitus maintain distress. Both therapies thus target cognition (albeit in ostensibly different ways – CBT through challenging the content of the cognition and MBCT through changing the relationship to the cognition). Importantly, they both encourage patients to stop avoiding tinnitus, and to approach it instead. MBCT and CBT are thus radically different from the treatments most often available as standard for tinnitus patients (which as we have seen tend to involve information, acoustic strategies, and relaxation) (McFerran et al., 2018).

By reducing avoidance, MBCT could be regarded as a type of “exposure.” MBCT also teaches individuals to relate differently to inner experience (particularly thoughts), and theoretically this should reduce emotional distress. Such reductions in distress may then create a condition in which habituation is further supported, in turn driving a “virtuous cycle” leading to greater tinnitus acceptance. Other known outcomes from MBCT (increased metacognitive awareness, reduced repetitive negative thinking, and self-compassion) are also likely to benefit tinnitus patients. This study aimed to explore what participants experienced in MBCT-t and how their understanding of what, how and why any changes occurred with treatment.

This study adopted qualitative methods which could assess contextual aspects of the intervention likely to affect treatment successful, and guide future research into mechanism. The participants’ voices articulate what MBCT-t is like and thus show new ways out of distress which can refine and improve the approach. Interpretative Phenomenological Analysis (IPA) is appropriate for exploring the lived experience of an individual when they are participating in a psychological intervention (Smith et al., 2009). Within this approach the knowledge and expertize of the researcher become an important part of the interpretation of the data and its meaning.

## Materials and Methods

The participants in this study had all completed MBCT-t as part of a Randomized Controlled Trial (RCT), and the full details of this procedure (including inclusion criteria) can be found in the article describing this trial (McKenna et al., 2017). This RCT has already shown that MBCT-t is effective in reducing tinnitus severity in chronic and distressed tinnitus patients, as well as reducing tinnitus-catastrophising and fear and increasing tinnitus acceptance, when compared to an active control condition (relaxation training). This study aimed to explore how and why MBCT-t might have this effect using a qualitative approach focused on the individuals’ experiences.

MBCT-t involved 8 weekly sessions of 120 min, and an overview of the treatment can be found in Supplementary Material. The majority of MBCT-t focuses on learning mindfulness and meditation and how to apply this to tinnitus. In addition there are specific references to the cognitive model: In week 2, Cognitive Theory is described and applications to tinnitus discussed, in week 4 the cognitive model of tinnitus is presented and in week 6 participants explore new ways of responding to challenging thoughts using mindfulness.

Participants completed MBCT-t (8 weeks of treatment). After this there was no additional treatment other than two follow-up sessions (at 1 and 6 months after treatment). Only once participants had completed their 6 months follow up were they given information about this interview study. Participants expressing interest in this study were invited to take part and provided full informed consent. Participants were only eligible for this interview study if they had completed MBCT-t as part of the RCT. The first nine respondents were included. Ages, gender, Social Economic Status, and tinnitus duration varied; the latter was of at least 6 months and all tinnitus subtypes were permitted (see Table 1). Names have been changed to ensure confidentiality.

**TABLE 1:** Characteristics of participants (anonymized).

	**Age**	**Gender**	**Tinnitus duration (months)**	**Tinnitus severity (pre-treatment TQ score)**	**Tinnitus severity (6-month follow up TQ score)**
Sarah	59	F	120	34	7^∗^
Kelly	52	F	74	56	47
Joe	67	M	120	28	12^∗^
Adam	58	M	360	52	13^∗^
Matthew	54	M	128	27	14^∗^
Peter	64	M	360	50	37^∗^
Sam	35	M	18	57	39^∗^
James	54	M	36	43	36
Damien	36	M	204	59	19^∗^

**^∗^Clinically significant change on TQ (11 point reduction).**

### Procedure

Participants were invited to take part following their 6 months follow-up and the initial nine respondents were interviewed. Interviews were based on a semi-structured interview schedule following guidelines for IPA (Smith et al., 2009). The first author (EM), one of the trial clinicians, conducted the interviews. The interview had two parts. The part reported here focused on their experiences and effects of MBCT, the other part focusing on their healthcare journey has been reported elsewhere (Marks et al., 2019). The interview schedule asked participants to reflect on their experiences of MBCT-t including the practices, teachings requirements, group setting, application of mindfulness to tinnitus and other aspects of their life, changes they noticed, external issues affecting their experiences and any longer term effects since completing the course. They were asked to reflect on how and why they felt MBCT-t had the impact it did and what they might say to others considering the course. An overview of the interview schedule is available in Supplementary Material. Ethical approval was provided by local UK NHS research ethics.

Interviews were transcribed verbatim by the lead author and analyzed with IPA, in line with the four-stage process of by Smith et al. (2009): (1) Interpretative reading of transcripts, notation of initial responses; (2) Identifying emergent themes; (3) Reviewing themes and mapping thematic connections; (4) Aligning sub-ordinate themes within super-ordinate themes. Cases were analyzed one at a time in this way, then across-case patterns were detected, creating a set of themes for the whole group. Repeated discussion and checking of themes and data by the second author occurred through an iterative process.

## Results

Four superordinate themes were identified: Two describing *what* changed: “Relating to Tinnitus in a New Way” and “Holistic Benefits,” and two describing *how* this was cultivated: “Connection, Kindness and Compassion” and “What supports engagement and change” (see Supplementary Material for a summary).

### Supraordinate Theme 1: Relating to Tinnitus in a New Way

All participants described a radical change in their relationship to tinnitus and how this reduced their distress. Some reported tinnitus reducing in volume or pitch, but this was secondary to their transformed relationship with tinnitus. Changes involved recognizing how their existing coping strategies (of resistance and attempts to control tinnitus) paradoxically exacerbated their difficulties, whilst experimentation with allowing, accepting and turning toward tinnitus reduced their suffering. They cultivated new ways of being with tinnitus by staying present, having more open, stable and flexible awareness, without fighting tinnitus, and reclaiming life.

*Sarah*: I wasn’t at war with the noise… That was the main bit…… in a way I’ve controlled it by not controlling it… at the start I knew I had to control it, because otherwise how am I going to manage it, and I didn’t know any different at the start of the programme. I thought, *I’m going to learn to control it*, but actually what you do is learn to let it go, and just if it’s there, it’s there.

#### Staying Present

Prior to MBCT-t, most participants reacted to tinnitus by striving to keep it away, through “*ignoring… putting on the TV… being busy hard at work*” (Sam). MBCT-t changed this, inviting a gentler approach of paying attention to one’s present moment experience and “*actually being aware of what’s inside you*” (Matthew). This improved and was seen as fundamentally different from distraction which was “*not deep enough… you haven’t developed any skills*” (Sarah). Mindfulness is “*awareness*” (Kelly) or “*noticing*” (Peter) for all participants except Joe, who found the attentional training actually improved his ability to distract away from tinnitus.

*Damien:* I don’t think it’s a distraction from your tinnitus because ideally you’re sitting there quietly, focusing. So I think actually I’m more aware of my tinnitus when I’m doing the mindfulness… focusing on the tinnitus is what separates that from doing any relaxation exercises.

Thus MBCT taught participants to stay present with all experiences, including difficult tinnitus. This was initially challenging, as tinnitus felt intolerably unpleasant. Time, repetition and the use of specific, tangible objects as the focus of attention (sights, breath, body sensations, the soundscape) helped with learning this skill. Participants felt less overwhelmed by tinnitus when they were able to keep refocusing attention onto one particular aspect of experience. This practice meant they were less caught up by repetitive negative thinking, challenging emotions and tinnitus “spikes.” Although effort was required to develop this skill, once learnt, it required far less effort than pre-existing strategies of avoidance and distraction and strikingly, this meant that many participants reported the novel experience of feeling peaceful and relaxed even when aware of tinnitus.

*Matthew:* I still get annoyed by things now and again, but things don’t fester any more, like they used to do… It’s like, let it pass… it’s almost stopping thinking too much, letting be… I wouldn’t say it’s sort of an aggressive or forced act… It’s not STOP, I’m forcing myself to stop now. It just like, okay, leave it… become aware of breathing, trees, birds singing, sky, whatever.

Growing awareness of one’s body helped with staying present, and this feeling of being “*more solid… functioning… whole*” was “*quite a revelation*” (Matthew) that gave participants the power to stay peacefully with whatever arose in each moment. There was a “*power of being aware of yourself and how you’ve got choices in how you deal with things*” (Kelly), which made participants more curious about their selves.

*Matthew*: (Mindfulness) brings a new layer of awareness and focus and peacefulness into all of it… your inner self maybe has got so many parts to it that you explore and certain things you do help you to explore certain parts of that inner self.

Joe understood the process of MBCT-t differently from the others, seeing it as distraction, but he still had a similar experience, noting that “*the effect of tinnitus was dramatically less*” during meditation because “*while you’re focused on something else you’re (not) worried by your tinnitus*.” This indicates how actively doing meditation matters more than understanding it. Mindfulness may have different functions for different people at different times; from switching attention between stimuli to expanding attention to include more stimuli.

Staying present with tinnitus challenged the distressing belief that tinnitus must be controlled in order to be tolerated. By observing their automatic reactions to tinnitus during meditation, participants realized that their habitual thoughts and behaviors actually increased suffering. Recognizing how “*your brain defaults to a pattern of behavior*” (Sarah) was liberating, because it meant new thoughts and behaviors could be tried out. Mindfulness didn’t stop default patterns from happening in the first place, but rather allowed participants to “*cast them aside*” (Peter) and choose a less reactive and resistant way of being with tinnitus. When awareness of tinnitus increased, it obviously provoked discomfort. But this discomfort was an essential part of learning how to relate to tinnitus differently. By noticing, acknowledging and allowing their discomfort without resistance, participants learnt how to allow themselves to be as they are, a kind act that helped them to heal.

*Kelly*: allowing… when I can bring it to mind… gives me permission, either to be angry, to be sad, to be accepting or just be without feeling anything necessarily…. Like a relief, a burden is lifted… You learn. You can only do that by allowing it. If you don’t allow it, you’ll never know what it feels like to allow it.

Participants described “open” awareness, a capacity to flexibly notice multiple facets of experience which created an expanded sense of self. This ameliorated tinnitus distress because they no longer felt so “trapped” by tinnitus, a common part of distress (Marks et al., 2019), because when tinnitus feels bigger than the self, it is overwhelming. Attention expanded around tinnitus, creating inner “*spaciousness*” (Damien).

*Damien: “*I guess I feel like I’m not thinking so much within my head. I’m focusing on stuff that’s outside of my brain… especially focusing on breathing and feeling the breath further down the body.”

MBCT-t is unique in its repeated invitation to purposefully listen to tinnitus with curiosity. This challenging new idea initially aroused irritation about paying attention “*to a lot of noise that I already know*” (Sarah). But “*listening to the tinnitus… being part of the daily landscape*” actually became “*one of the biggest changes*” (Sam). Participants realized that “*what I’m hearing in my own head is not the only thing to be heard*” (Kelly) and tinnitus shrank from an all-encompassing monster to one small part of a larger self. Taking “*a step back*,” participants could “*see the bigger picture*” (James): Tinnitus shifted from being the index problem, to being regarded “*more globally… a symptom of other things*” (Matthew). In contrast to traditional tinnitus treatments, MBCT offered new ways of “thinking about” and “being” with tinnitus, by learning how to stay present with all experience.

#### Equanimity (Allowing and Letting Be)

By paying close attention participants recognized the ever-present flux of all experience, including tinnitus. Understanding that tinnitus (and all difficult moments) eventually pass on their own, reduced how much participants felt they had to strive fix tinnitus. Catastrophic thinking was replaced by acceptance.

*Sam*: “accepting that… it probably is always going to be there… just because it’s screaming really badly this morning, doesn’t mean you’re going to notice it all day long.”

This equanimous attitude of allowing tinnitus to be as it is, is purposefully fostered in MBCT-t, with participants experimenting with what happens when they treat pleasant, unpleasant and neutral stimuli with equal respect. It initially required courage to “*to get up close to it*” which was “*quite scary*” (Sarah), but listening to tinnitus was surprisingly “*okay*” (Damien). Tinnitus was less terrible, and more changeable than expected. Paradoxically, paying attention to tinnitus without seeking to change it, changed the experience of tinnitus.

*Adam: …*even the negative side is just a feeling… if you can treat everything with the same respect, the good and the bad… it’s going to go in a certain amount of time.

Attending to tinnitus meant participants could see their reactions more clearly and gave them an opportunity to update their assumptions about it. They realized that fear of tinnitus is different from the reality that it is “*not yesterday’s tinnitus, not tomorrow’s tinnitus, but now*” (Sarah). The reality of tinnitus was easier to cope with than the fear of it, and paying attention to it unexpectedly engendered calmness. This was not because “*mindfulness per se*” is relaxing (Sam), but because by learning to “*go with the flow*” (Joe) they were “*less likely to get flustered*” (Damien) by tinnitus. With practice, participants trusted that experiences would come and go of their own accord. Sarah described how reminding herself that “*it’s already happened*” allowed her to stop resisting unpleasant experience. This required neither resignation nor blind trust, but a growing clarity grew of understanding what aspects of experience one can and cannot influence.

*Sarah*: I don’t sit and worry…. I know what I can do about it and I know what I can’t do, so why am I giving it this extra energy? It’s already happened… I have no control… It doesn’t mean it doesn’t get me down… but it will sort itself out.”

Participants were striving less and no longer “*at war with the noise*” (Sarah), choosing not to “*expend much energy worrying or getting cross*.” For some, tinnitus itself improved, Sarah stopped having a painful experience of tinnitus “*fireworks*,” Damien reported reduce tinnitus “*volume (and) pitch*.”

*Adam*: It hasn’t affected the tinnitus… It’s the way I think about it and the way I deal with it… knowing that in just a few moments it won’t be as bad… you have to accept that it’s there, you have to.

The swiftness and breadth of acceptance varied and Sarah noticed in just 4 weeks that “*I haven’t even thought about it today*” and after a few months that “*I don’t think I’d class myself any more as having invasive tinnitus.*” Those with smaller and slower changes benefitted too (e.g., Joe may have still had intrusive daytime tinnitus but this disappeared at night). Overall, most participants felt less limited by tinnitus, confident that even if they had a “tinnitus spike,” they would cope.

Damien … I’ve been pushing myself to do more things, so I’m having a better life really… going to concerts… flying… I think it just gave me a bit of a kick you know.

MBCT-t was not a panacea, and many participants felt resignation as well as acceptance or “*the right mental attitude*” (Sam). Most still wished for a definitive “cure.” Yet participants saw the big advantage of mindfulness being it’s ability to empower them, offering a tool to “*take away*” (Damien).

### Supraordinate Theme 2: Holistic Benefits

MBCT-t was associated with many additional benefits. Distress reduced as negative thinking became less tyrannical, associated emotions (depression, anxiety, frustration, anger), difficulties (stress, insomnia, interpersonal conflict) and avoidance behaviors eased. Enhanced wellbeing developed across life domains with positive states of gratitude and joy flourishing.

*Sam:* …mindfulness in general would appear to me to be a really beneficial thing in real life… some of the people who come through the course naturally lean towards depressive tendencies…. I kind of just got the sense of there being more than the tinnitus that people are being helped with.

#### Reduced Distress

Many participants had long-standing anxiety, depression and stress which MBCT-t helped. Adam saw himself “*as being treated for depression*,” Sarah became less “*jumpy*,” Matthew used mindfulness to “*de-stress*” and Damien “*to manage anxiety.*” MBCT-t is known to reduce psychological distress in tinnitus (McKenna et al., 2017, 2018b), and participants related this to reduced repetitive negative cognition or “*almost stopping thinking too much*” (Matthew). They learnt to identify and disengage from negative thoughts which reduced negative reactions to events spiraling out of control. Participants had more “*choice*” about how to respond to a situation and “*rather than just going ‘poof’ straight away”* (Kelly), they could slow down, choose a new perspective and response. This was particularly profound for Sarah, who’s recurrent suicidal (“*invasive*”) thoughts reduced in frequency, and even when they arose she observed them without being overwhelmed.

*Sarah*: the rest of me has benefitted… You can’t catastrophize over everything else but manage it with your tinnitus. So it’s holistic… the big thing is the not catastrophizing…

#### Enhanced Wellbeing

MBCT led to more calm, relaxed or tranquil states, offering an “*inner support*” (Kelly), with “*rewards in itself*” (Matthew). This arose naturally, something that “*permeate(s) a lot of things… it’s a general, more relaxed approach to things*” rather than through trying hard to create a state of relaxation. This coincided with growing equanimity across life domains. By learning that one “*can’t prevent things… you just have to let them go*” (James) they learnt to relax control. A growing sense of peace was a significant experience for several participants. It is important as many tinnitus sufferers fear that constant tinnitus will prevent them from ever having “peace and quiet” again. Yet experiences in MBCT-t refute this idea, showing it is possible to have peace even as tinnitus continues. Perhaps peacefulness arises not when there is an absence of *sound*, but when there is an absence of *war* (when the fight with tinnitus stops).

Positive behavioral changes were commonplace, supported by greater energy reserves, better mood and growing commitments to self-care. These changes were profound, enriching and “*liberating*” (Kelly) as “*horizons opened*” (Sam). Feeling calmer benefitted participants’ interpersonal relationships: Matthew’s wife noticed he managed anger better by “*not to get to that tipping point*”*;* Adam noticed less family conflict and Kelly reported calmer interactions at home and work. Again Joe’s experience was different, because he felt his tendency to keep emotions “*bottled up*” meant others wouldn’t observe changes in him. Improvements in sleep were reported, with Joe describing that he would “*worry less at night…(and) fall asleep sooner*.” Damien able to “*sleep without (tinnitus) disturbing*” him and Peter replacing sleeping pills with meditation.

Gratitude, appreciation and joy arose during the course, significantly contributing to wellbeing. This was interwoven with other aspects of mindfulness such as paying attention, being less busy and feeling more equanimous and connecting to the world, through “*listening to what is going on around you… stuff you don’t usually have time for*” (James). At first this required making intentional decisions notice pleasant events, and appreciate fleeting beauty that previously would have been missed. In noticing joy, experiences of struggle and distress reduced.

*James*: You can appreciate things more… whether it be your pet cat or the squirrel running across the world… a good day or a bad day… if you take time you can appreciate it a bit more.

As mindful awareness became more integrated into their lives, joy and gratitude arose more spontaneously, with gratitude springing from connections with others, seeing kindness and generosity in other people. Simply paying attention transformed experience as “*it just melts everything away for a moment*” (Kelly).

### Supraordinate Theme 3: Connection, Kindness, and Compassion

MBCT-t deepened connections with others and the self, particularly through recognition of shared suffering and shared humanity, which generated kindness and compassion toward those suffering. The process of MBCT-t involved increasing sensitivity to suffering and a wish to alleviate or prevent it in oneself and others. This aligns with other group interventions, although specific attitudes of kindness and compassion appeared to be fostered particularly by the foundational attitudes of mindfulness.

#### With Other People

The community that arose from the group nature of MBCT-t was an essential part of the process. All participants spoke of the value of being with other people with tinnitus, benefitting from consolation, support, motivation, education and insight. Connection was immediate and instinctive, and the group reduced isolation and loneliness, as nobody was “*singled out as only person who has to suffer*” (Matthew). Shared suffering transcended other differences, and everyone felt that the others would understand and accept their distress. This shared connection was an essential element of the therapeutic process, indicating the benefits of offering tinnitus treatments in a group format.

*Kelly:* there’s just an instant connection, even if that person is completely different to you in all other respects, they just know, you know, it’s an unsaid thing… powerful… in some respects it could be quite healing… collective of sharing… makes you feel like you’re not the only one.

MBCT-, with its’ focus on skills-learning supported engagement and connection, possibly as it was easier to be in a “*class*” rather than a “*therapy group*.” Sharing experiences was “*enlightening and strangely comforting*” and aided learning through “*knowing that other people are maybe coping*” which offered hope they could cope too. Comparing one’s own suffering to others’, greater struggles put one’s own tinnitus in perspective, reducing the sense of overwhelm and stimulating gratitude; both powerful antidotes to negative cognitions. This included a teacher’s disclosure of personal experience with tinnitus because “*empathy could be more powerful sometimes than anything else*” (Kelly).

*Peter*: There’s a lot of people with worse things you know… to see someone worse off, at least it makes you more aware of what people have to cope with… thinking about other people’s problems rather than concentrating on your own… other people can maybe cope with worse than you… that should make you able to cope with yours.

Empathy led to compassion, particularly for participants struggling with greater stress (busier lives or worse tinnitus), and from this a real wish for their wellbeing.

*Adam*: ‘The only person I thought who had it worse than me… she did seem to improve as the weeks went on… I really really do hope that she improved…

Participants learnt from each other in different ways, including observation (such as seeing “*how they were sitting*” (Damien)), listening to their comments (“*negative or positive*” (James)) and hearing the *inquiry* (where teacher and participant discuss experiences together). It was very important for the group to welcome skeptical and negative reactions, probably because it demonstrated that mindfulness was being used authentically. This welcoming of all experience is a key aspect of the MBCT-t group approach (Crane et al., 2010).

*Adam*: I can understand now how people will be skeptical, but it doesn’t matter, because I know, I’ve seen it work… my own experience but from other people’s too… the group is… informative for everybody.

Participants were motivated by “*how much work*” others put in, feeling “*a responsibility*” to do the same. For Damien, this was “*one of the main reasons I started timetabling my mindfulness*” and for Sarah, hearing how people used their own “*personalities and skills to get to grips with the mindfulness*” helped her think about what was best for her. Unlike some group therapies which might focus more stories about the past, MBCT-t supported learning by focusing on how to engage in particular skills and practices in the here and now.

Connections with the service and the teachers were essential for trust, engagement, commitment, hope, and embodied learning. Equally valued were professional expertize (in mindfulness, tinnitus and psychology), and personal attributes (experience of tinnitus, commitment to mindfulness practice, and embodied attitudes of patience, non-striving, non-judging, acceptance and compassion, e.g., Kabat-Zinn, 1990). Being “*professionally run*” (Sarah) within the NHS and specific for tinnitus, with good organization and delivery meant participants felt safe, and could feel both trust and skepticism at the same time. Feeling safe in the group gave participants more confidence to try out new and challenging practices in the belief that they might help. As Adam described, “*(your service is) the best… if you can’t do something then not many people can*,” and there was “*less potential for anything negative to happen*” (Damien). This is important when considering the context of a growing mindfulness industry where levels of regulation and supervision vary enormously, and when research has shown that expectations of therapy correlate with outcome (Greenberg et al., 2006).

*Damien*: …very well set up and appropriate… you’ve adapted the course … very clear about objectives… The course material was really good… really well organized… an NHS project, properly funded.

Participants learnt how to be mindful by observing their teachers’ embodiment of mindfulness, as reported elsewhere (Crane et al., 2010). This included teachers balancing gentle permissiveness with structured discipline. For example, through “*being able to sit or lie down wherever*” (Peter) or having meditations guided “*in a sensitive way*” (Matthew). Participants learnt treat themselves in the same way, “*to take what you can and leave the rest*” (Kelly), allowing them “*to work out their own way*” (Adam). Similarly, the teachers’ attitude of “*completely non-judging things*” was anathema to participants’ habitual “*judging oneself as substandard*” (Kelly), and feeling “*looked after*” (Damien) and accepted by the teachers was the ground upon which participants began to develop compassion for themselves.

*Kelly:* (the) teacher… always been very gentle and very allowing… when I can bring it to mind it gives me permission (to be as I am).

Connection and compassion seeped out of relationships within the group into everyday life. Sarah found that her natural empathic nature became increasingly infused with kindness, leading to more compassionate responses to challenging situations. Thus if conflict arose, Sarah felt “*less under attack*” and she had the psychological space to respond wisely. Sam found that MBCT-t increased “e*mpathy and my psychological understanding of other people’s behavior, so I’m more connected*,” Adam found it “*makes you want to be a better person*” and James felt that kindness was a “*knock on effect*” as he developed more “*empathy to people, animals, etc*.” Matthew felt “*more empathetic, sympathetic*” toward “*everything: people, animals, the world*” (including himself). Sarah felt connected to even strangers with tinnitus, and marveled at her deep wish for everyone to receive the help she was receiving. In opening to human suffering, compassion bloomed:

*Kelly: …*like if someone’s running for a bus and I’ll think “ah I really hope that bus waits for them” whereas before I might have been “go on, drive off” (*laughs*).

Feeling kind toward others was supported by the ability to stay present and be less caught up in automatic thinking. Kelly described how she started to notice how she had automatic negative thoughts about other people that made her feel disconnected from them (“*why is he doing that*”). With mindfulness, she stopped being caught up in negative stories and chose instead to notice other people more neutrally (“*oh so and so is doing that*”). She began to feel more curious about others, more willing and able to offer them attention (“*oh well that person is really wanting to know about me and I want to know about them*”) and eventually more likely to offer them kindness; for example, she took on a pastoral role at work “*to give something back*.”

#### With One’s Self

MBCT-t involved offering compassion “*to me as well*” (Kelly). Most simply, paying attention to oneself and allowing oneself to be is an act of self kindness. This applied to neutral stimuli, such as noticing the breath with an attitude of “*gentleness*” (Sarah) and to aversive stimuli, such as tinnitus, recognizing it as part of oneself and allowing it to be present. These shifts in attitude toward the self and inner-experience laid the groundwork for bigger life-changes.

*James*: you’re giving yourself that time which in everyday life you don’t have… to actually let things go, breathe easy, express themselves, I think is very important.

Self-kindness changed the habitual language of the “inner critic.” The old voice that might say “*oh now look what you’ve done” or “moron”*, was replaced with a kinder voice saying “*what you did then you thought was the right thing to do*.” Participants learnt to forgive themselves for making mistakes, which made making mistakes and asking for help less threatening (as it was less likely to result in a tsunami of inner-judgement). In realising her difficulties were shared with other humans, Sarah could ask for help without feeling ashamed. In feeling kinder toward himself, Matthew stopped caring about others’ judgments, and could do what he wanted, not what he felt others expected of him. Self-compassion could elicit fear, particularly when it threatened high-standards or where over-permissiveness might risk a complete collapse of boundaries: “*what if I allow too much?*” (Kelly). This is an important issue to discuss in the group, as participants find the right boundaries for themselves: “*you learn – you set your own limits which you can only do if you first learn to allow.*”

*Matthew:* it’s about treating yourself well, which you know it’s quite easy not to do. It’s quite easy to punish yourself, to criticise yourself and not actually congratulate or reward yourself… (now) I tend to sort of do what I feel like is right for me, not what I feel like what I’m supposed to be doing.

Mindfulness helped participants to be kinder overall, which in turn freed them up to think differently and move out of familiar patterns of criticism.

### Supraordinate Theme 4: Factors Supporting Engagement and Change

Engagement with MBCT-t and sustained practice led to change, and depended upon having an open mind, tempered by welcoming skepticism. Various internal and external supporting factors supported included motivation, personal characteristics, stress levels, time, help and appropriate practices. MBCT-t required perpetual re-balancing of hope with realism and gentleness with discipline.

All interviewees had been committed enough to MBCT-t to complete it, although enthusiasm and skepticism varied. Most had begun the course without knowing what to expect, but reported prior experience of complementary therapies, indicating an open mindedness toward “alternative therapies” which probably helped engagement. This included cranial osteopathy, acupuncture, Tai-Chi (Sarah, Matthew, Kelly, Damien), meditation (Adam, Peter), psychotherapy (Kelly, Matthew) or Buddhism (Sam).

*Damien*: I’m sceptical about alternative therapies … but I found it a quite pleasant experience when I got around the slight weirdness of it… Previously I’d had a good experience of an alternative therapy route. I didn’t know anything about mindfulness prior, so that’s what I thought it was.

MBCT-t appealed as a non-invasive intervention for something which medical or surgical routes had failed to help (Marks et al., 2019). Several people expressed preferences for a therapy helping them to “*get inside (their) head and figure out a way of working with it”* (Kelly), rather than a “*cure*.” This is an important point, as participants here recognized that there is no “cure” available to “silence” tinnitus. This is at odds with a growing cultural narrative which claims that medical interventions will always be the preference for everyone.

Skepticism and open-mindedness came together, and both facilitated change. Open mindedness led people to engage, and the permission to express skepticism prevented people from feeling alienated. Adam feared the Buddhist origins of mindfulness might be too spiritual, but decided to “*see what happens*.” James, wanting a cure, was “*apprehensive… didn’t think it would benefit me*” and only attended because he had committed to it. Realistic expectations were helpful, as Sam stated, he would recommend it to anyone unless they were “*dead set on treatment that was tantamount to a cure*.”

*Sam*: my wife was doing some research… I wasn’t sure but I thought I may as well… part of me thought that probably it isn’t my thing and it probably won’t do any good, but actually for me, I was surprisingly open minded to it…

Practical factors made important contributions to the experience of MBCT-t, and the biggest one was practice. Commitment and patience were required to allow for slow and “*gradually revealing,”* changes, because one *“couldn’t expect a miracle too quickly*.” Meditation practices tended to be difficult at first, becoming more enjoyable and easeful with growing familiarity.

*Damien*: I found some of it overwhelming because it’s not something I’m really used to… I got more used to it over time. Towards the end I was enjoying… it, just became a bit more normal.

The structured nature of MBCT-t helped, and attending weekly sessions preserved momentum and learning, because “*the group made mindfulness easier for me than doing it myself*” (Sam). Participants valued having an accessible and welcoming location supported by appropriate equipment (cushions, mats etc.). Background noise in the room (air conditioning), irritated some, exacerbating tinnitus and hearing loss, but consoled others as it “*partially masked the tinnitus*” (Damien). MBCT-t groups should consider advising use of appropriate background noise for classes and home practice.

A unique aspect of MBCT-t that supported change was the focus upon *sound and hearing* meditation and inquiry. This was described as “*the most important*” practice (Kelly), particularly attending to the entire soundscape (not just to tinnitus) and noticing sound directly (rather than thinking about sound). This was very challenging at first, as sitting still with tinnitus usually led to an initial increase in its’ prominence. Participants needed commitment and courage to continue, and eventually the benefits unfurled. Meditation became easier, as practice led to benefits which led to more practice. For example, Sarah who had a strong home practice found mindfulness worked “*quickly… not hearing the tinnitus as quickly*,” keeping her very engaged.

*Damien:* it was important to challenge myself… the more I practiced, the more I got out of it. I was determined to give it my best shot… practicing outside meant that when I was doing it there it didn’t feel so foreign.

Participants re-learnt how to listen, with more expansive awareness of multiple noise: “*voices, traffic and my tinnitus*” (Peter). This helped them to reconnect with the joy and beauty of sound, particularly in pleasant or natural environments.

*Kelly*: there’s lots of birds in our garden, and I love that because I suppose that when I’m listening to that… I’m also aware of all the other sounds.

This was not easy, and simply guiding sound meditation was insufficient. Participants wanted repeated, explicit teaching and discussion about how to mindfully attend to sound and how mindfulness applies to tinnitus. Watching an MBCT-t alumni speak of his experiences on film helped with this somewhat.

*Sam*: Maybe there could have been more to link the mindfulness activity specifically to the tinnitus… treating us like idiots and explaining step by step, this is how it can affect your coping with tinnitus.

The more serious aspects of meditation practice could be challenging, and integrating lighter and more humorous attitudes was sometimes helpful, particularly in encouraging gentleness, as seen in Kelly’s playful approach to sound meditation.

*Kelly* ‘It’s a shift I remember… when I’m in the shower I will always try to listen to the water and I might go to this place where I’m going ‘water, tinnitus, water, tinnitus’ (Laughs).

The demands of daily life made practice difficult, but priorities changed as the benefits were experienced directly. Adam, noticing how his stress increased if he stopped practicing, realized that “*if you don’t do it, it doesn’t work.”* On seeing benefits, some participants reduced their practice but still felt confident knowing that it “*worked before… should be able to work again*” (Damien). Practical strategies such as daily schedules reduced cognitive loads of remembering and deciding to practice, which helped.

*Damien*: other people were saying they were practicing and getting a lot out of it … initially I wasn’t dedicating enough time to it… I had to be quite strict about timetabling it into my day… you were investing in me… so I felt like I had a responsibility to the group.

People saw a correlation between frequency and duration of practice and quality of their life, particularly when supported by the ongoing sessions.

*Damien*: I had a better quality of life in that period when I was regularly practicing mindfulness… the most at peace and the best I felt were really deep, long meditations.

Clearly, induction to MBCT-t must ensure participants have the time, energy and will to commit to practice. This includes learning to meditate at a point in their life where there is space to practice, because “*meditation is easier… if you’re in a frame of mind to meditate… you have to be in the right mood*” (Joe). It also includes cultivating an attitude toward practice as something fundamental, part of self-care, health-care and an opportunity to learn something new, rather than just another burden.

*Sarah:* commit to all the work… not to see it as a chore, as I see it as an opportunity to take control, to retrain the brain.

Flexibility in practice was important, and each person discovered how to fit mindfulness to their particular circumstances. This made the shorter and more informal practices popular, including mindful awareness of activities such as “*laying in the bath*,” *“feeling the ground under my feet,”* (James) or existing exercise routines (Peter, Matthew). Portable practice, such as a few minutes on the daily commute “*could be more powerful than doing something for 30 minutes”* (Kelly).

Thus the type, intensity and regularity of practice varied across individuals, with learning occurring in each participant differently, but in equally valid ways. Sarah explained the importance of using “*whatever their learning style is to help them with the meditation*,” and how teachers must be able to support participants to use their particular skills and characteristics to “get to grips” with mindfulness. This includes creative approaches to standard exercises such as breath and body awareness.

*Sarah:* As a visual person… I came up with… like when you see mercury sliver, fluid… I had that image in gold… I even used it to move around the blood… because the breath on its own I just couldn’t get a handle on that.

Unpleasant and aversive experiences were common, with descriptions of the eating meditation as a “*waste of time*” (James) and imagining breath traveling through the body, as “*so silly*” (Joe). Here an open mind and an option to try other exercises helped keep people engaged. The stories and poetry weaved throughout MBCT-t were “*refreshing… different ways of looking at it all*” (Matthew). However, the CBT specific exercises were rarely recalled by participants unless prompted. These psychological principles “*made sense*” (Matthew, Sam) but didn’t seem as critical to learning as other aspects of the group.

## Discussion

Four over-arching themes emerged from the IPA: *Relating to Tinnitus in a New Way; Holistic Benefits; Connection, Kindness and Compassion; and Factors Supporting Engagement and Change.* These resonate with qualitative research across other MBPs. The discussion focuses first on how MBCT-t applied to tinnitus followed by some reflections on more general experiences.

### How Did MBCT-t Apply to Tinnitus?

Every participant reported a transformation in their relationship with tinnitus. The shift from “being at war with it” to “allowing it” was the core process and outcome shared across the group. A few people reported improvements in tinnitus volume and pitch, but this was not universal. Participants explained how they became aware of automatic thoughts (rumination and catastrophizing) and coping behaviors (resistance, suppression, control) and discerned how these were unhelpful. MBCT-t taught them the theory and skill to relate differently to the experience, by turning toward and allowing tinnitus, without getting caught up in reactive narratives or behaviors. They described making significant efforts to apply mindfulness to the present moment, in an intentional, patient, friendly, trusting and equanimous way, with a view to reducing their suffering and enhancing wellbeing (e.g., Kuyken and Feldman, 2019).

Mindful awareness of tinnitus involved sustaining attention in very different ways from their previous fear-based selective attention and monitoring relationship with tinnitus. This new attentional style involved less cognitive and emotional reactivity. Participants could be purposefully aware of tinnitus without also engaging in catastrophic narratives, a need to make things different or feeling emotionally overwhelmed. This process could be seen as involving “exposure” to noxious stimuli (tinnitus) through the dropping of avoidance and safety-seeking behaviors. The development of meta-cognitive awareness through mindfulness may have led to the development of greater emotional stability which in turn could have supported habituation and tinnitus acceptance. Mindfulness also involved attitudes of gentleness, kindness and permissiveness, potentially enabling acceptance of tinnitus, rather than just tolerance of it, as indicated in other physical health conditions (Carlson, 2012).

Attentional flexibility increased, including the ability to refocus away from, or expand attention around tinnitus. Thus participants were not repeatedly “captured” and “overwhelmed” by tinnitus. Crucially, this did not mean mindful awareness involved refining existing avoidance or management strategies. Nor did it mean resignation or just “learning to live with it” as so many had previously been told to do (Marks et al., 2019). Instead, participants developed a new, acceptance-based perspective on tinnitus. They saw how striving to control tinnitus was often futile and counterproductive, and that the best way out of suffering was to let go of the struggle. By “allowing” tinnitus, they saw that even the worst moments always pass, without them having to do anything to make this happen. This meant they stopped engaging in fear-based catastrophic thinking, and instead chose a more equanimous attitude. Even the most skeptical participant (Joe) learnt how to “*go with the flow*” more often. This new perspective on tinnitus sits comfortably with the definition of mindfulness involving an ability to sustain and switch attention whilst inhibiting secondary elaborative processing.

Sound and hearing meditation were key to change, and although challenging at first, with encouragement and explanation about applying mindful awareness to tinnitus, this critical aspect of MBCT-t helped participants to develop a new relationship with the noise. MBCT-t does not involve learning any particular “acoustic strategies” (such as the “masking” or “partial masking” that characterizes sound therapy), the only acoustic strategy advised in MBCT-t is to encourage participants to approach and allow tinnitus, and to reduce their reliance on sound used for partial masking or distraction.

Of further importance were the location and make-up of the group and expertize of the teachers. Sharing experiences with other tinnitus-sufferers contributed strongly to the therapeutic nature of the intervention, as did having mindfulness teachers with expertize in tinnitus (personal and professional), based in an audiology center. This finding indicates why MBPs may be most effective when designed, tested and delivered in appropriate settings, including the integration of such programmes into holistic care. A biopsychosocial approach to health and illness is advised in tinnitus (McKenna et al., 2018a), and with clear evidence that MBPs are effective in tinnitus (Rademaker et al., 2019) clinical services must make these accessible to patients in the most effective contexts possible. This could include employing psychologists and MBP teachers in audiology services, or even training audiologists to deliver MBPs or work alongside mindfulness teacher, providing an audiological perspective within standard MBPs.

### Contextual Factors

The stories of these individuals accentuate how compassion and kindness are integral to both the process of and outcomes from MBCT-t. This particular MBCT-t course included explicit teaching on kindness in week seven (loving-kindness meditation and discussion about compassion). Participants explained how the course allowed them to offer themselves time and space to simply be, without judgment or alteration, including in response to tinnitus. This chimes with broader literature which indicates how increases in self-compassion could be one mechanism of change in MBPs (Kuyken et al., 2010). Self-compassion arose with compassion for other beings, through the recognition of shared human suffering, which allowed perspective taking and healing.

The related constructs, appreciative joy and gratitude, were also present across the different themes, emerging from MBCT-t as perspectives shifted. This included seeing comparing one’s tinnitus to the larger context of human suffering and being able to appreciate what aspects of life one is grateful for. This didn’t negate the pain of tinnitus, but did reduce negative cognitions and create opportunities to notice and connect with many positive aspects of life that are not limited to tinnitus. MBCT-t explicitly encourages gratitude practice through the use of a “pleasant events diary” in week 2. More recent developments in MBCT-t now include specific gratitude meditations.

MBCT-t involved the creation of a community, which in turn was a hugely therapeutic experience. This is seen in other MPBs (Williams et al., 2011). Developing this community didn’t require participants to share long histories about their personal lives and tinnitus, and in fact seemed to flourish through the shared experience of learning a new skill. The knowledge that one would be understood and accepted, and traveling together into new territory was enough to build strong connections across the group, which in turn supported other changes. This finding may indicate how tinnitus support groups will be more effective when the group has a shared goal or learning outcome, rather than simply involving reflection on experiences.

As with most MBPs, benefits extended beyond the primary concern, with improvements in depression, anxiety, stress, sleeping difficulties and interpersonal conflict. This is an important finding because such comorbidities in tinnitus are common (Baguley et al., 2013) and treatment that can ameliorate these will benefit patients.

This study shows how important it is for participants to find their own way through MBCT-t, and for teachers to balance discipline and structure with flexibility, warmth, a light touch and humor. Allowing, acceptance and compassion will flourish only if the individual can apply this to their own practice, experimenting with different postures, practices, learning styles and being able to express understandable skepticism, pain and suffering. All participants benefitted from the group, but in different ways (although Joe may have regarded mindfulness differently from others, he still benefitted). This links with the theory of mindfulness that one’s *intention* in the practice will probably shape what one learns. Teachers therefore have a pivotal role in shaping the intention of participants in a way that will bring the most helpful learning to them. Learning, however, did not come only through didactic pedagogy but was dependent upon how the teachers modeled and embodied attitudes and approaches. The ability to teach MBPs in this way requires high-level training alongside sustained personal practice, so MBCT-t will require services to invest in their staff sufficiently. This poses real challenges in current healthcare systems, but the positive outcomes from MBCT-t in the long-term (here at 6 months) indicates how such investment may pay for itself by reducing the “revolving door” of healthcare so many tinnitus patients experience (Marks et al., 2019). The benefits from MBCT-t will be long lasting because they are associated with profound changes in people’s attitudes toward and relationships with tinnitus and life more generally, something that current standard treatments fail to offer.

### Strengths and Limitations

Strengths of this study are its novelty; no other studies have explored how tinnitus patients experience MBCT-t and these findings offer clear routes for future research and clinical practice. The IPA approach conducted by an experienced clinician offers a significant depth of insight which is valuable in understanding processes of MBCT-t. Although only 9 people were interviewed, these came from two MBCT-t groups and reflected a range of ages and genders. Limitations of this study are that only committed participants (completing MBCT-t and 6-month follow up) were included. However, only 2 people overall (of 39) dropped out of treatment, so this is a minor issue. All participants were Caucasian which may limit extrapolation to a more diverse sample. The age range of those interviewed was 35–67 years, so extrapolation to younger or older patients samples may also be limited (although theoretically we do not envisage this as having a large effect on the processes described, particularly as age was not found to moderate outcome of MBCT-t in the original RCT). Tinnitus subtyping was not conducted as part of this study, and future research could explore whether this has an impact on treatment outcome. There is a risk of bias from the interviewer also being the trial therapist, but awareness of this was accounted for in the analytic approach, participants were willing to discuss their experiences at length and were prompted to report on challenges and negative experiences.

### Clinical Suggestions

Table 2 offers guidance for clinicians working with tinnitus sufferers and indicates how MBCT-t could be developed in the future.

**TABLE 2:** Clinical suggestions for applying mindfulness to tinnitus.

Stop fighting tinnitus, stop being “at war” and instead and turn toward it.	Mindfulness is not a “cure” to silence tinnitus, nor a way of getting better at “ignoring it.” It is based on the evidence that fighting tinnitus makes it worse. Conversely, allowing tinnitus to be present and even turning toward tinnitus will alleviate suffering. Paying attention to tinnitus without judgment or attempts to change it will lead to a new relationship with tinnitus where it becomes less intrusive and problematic. This can be done by purposefully listening to tinnitus as part of the soundscape, but without thinking too much ‘about’ it.

Develop mindful awareness	Standard mindfulness meditation practices (such as focusing on the breath, body, thoughts, emotions and movement) develop one’s ability to remain present with experiences. As this ability grows, it can be applied to difficulties such as tinnitus in a helpful way.

Connect with others	Connect with other people who have or understand tinnitus and building a community, such as a therapeutic group. This helps one to feel less alone and isolated. It may be more helpful for the group to focus on learning something new together, rather than focusing only on tinnitus.

Take a broad perspective and practice gratitude	See tinnitus in the bigger context of all human suffering, and gain perspective on the fearful and catastrophic thoughts that might exist about it. Try experimenting with noticing other aspects of life which one can be grateful for.

Develop kindness and compassion for yourself and others	Experiment with approaching all experiences (including tinnitus) with a kinder, more gentle and friendly attitude. Be kind to oneself by engaging in activities that are fun or pleasant. Try practicing Loving-Kindness meditation and offering kindness to other people.

Balance discipline with permissiveness	Learning mindfulness requires a balance of discipline and permissiveness, and regular practice is vital. Sustaining practice will involve finding ways of practicing that suit the individual and their current life circumstances.

## Conclusion

MBCT-t brings people to a long-lasting, radically different, and more helpful relationship with their tinnitus. They shift from being “at war” with tinnitus to “allowing it to be.” Multiple processes interweave to lead to these changes in perspective and attitude, including developing mindful awareness, being in a group, embodied teaching, compassion, gratitude and having an open mind. MBCT-t can be successfully applied to a tinnitus population, in a way that changes the factors that maintain tinnitus-related distress (catastrophic thinking, selective attention and anxiety). MBPs should continue to be developed and utilized to offer increasingly effective tinnitus treatments, and made available to tinnitus patients as part of the standard care which is still frequently limited to information, acoustic strategies and relaxation.

## Data Availability Statement

The data supporting the conclusions of this article will be made available by the authors, without undue reservation, to any qualified researcher, to be accessed at University College London Hospitals or the University of Bath.

## Ethics Statement

This study involving human participants were reviewed and approved by the Camden and Kings Cross NHS Ethics Committee. The patients/participants provided their written informed consent to participate in this study.

## Author Contributions

EM designed the study, conducted and transcribed the interviews, led the analysis, and wrote the manuscript. PS and LM contributed to the analysis of the interviews, in line with standard IPA methods, and commented on the manuscript. All authors approved the final version of the manuscript.

## Conflict of Interest

The authors declare that the research was conducted in the absence of any commercial or financial relationships that could be construed as a potential conflict of interest.
