# Supplementary material for: I Wasn’t at War With the Noise: How Mindfulness Based Cognitive Therapy Changes Patients’ Experiences of Tinnitus
Source: Front Psychol. 2020 Apr 17;11:483. doi: 10.3389/fpsyg.2020.00483 (PMC7182032; doi:10.3389/fpsyg.2020.00483)
Supplement: Supplementary file 1 [file Data_Sheet_1.docx]

**Supplementary Material:**

1. **Summary of Interview Schedule**
2. Can you tell me about your experiences of the Mindfulness Based Cognitive Therapy course?
3. How did you feel about the requirements of the course?
4. Did attending the MBCT course and doing the mindfulness practices have any impact upon you? In what ways?
5. Can you tell me about any specific aspects of the course you think led to changes?
6. Can you tell me how you found being in the group setting?
7. Did you find that mindfulness could be applied to tinnitus?
   A) If so, how and why do you think this happened?

B) If not, how and why do you think this was the case?

1. Can you tell me if the mindfulness approach helped or didn’t help you with any other experiences?
2. If so, can you give me an example?

B) If not, can you tell me about why you think this is?

1. Was there anything outside of the classes that helped or hindered you learning or using MBCT?
2. Do you still practice things that you learnt in the class? If so, please describe your experiences of it.
3. Over the last 6 months, since finishing the MBCT course, have you noticed any changes in your life which you think are due to the course?
4. *Dependent upon answers so far:*

*EITHER*

It sounds like you have had a largely positive experience of the MBCT. Can you tell me why you think this has been so helpful for you?

*OR*

It sounds like you have not found the MBCT to be especially helpful. Can you tell me why you think this has not been very helpful for you?

1. How do you feel about your tinnitus now, and in the future?
2. Do you have anything to add that to help us to understand your experiences?
3. If someone with tinnitus was trying to decide whether they should attend this course, what would you say to them / what three tips might you give to them?
4. **Overview of Mindfulness Based Cognitive Therapy for Tinnitus intervention**

The intervention was based on the standard MBCT protocol for depression (Segal, Williams and Teasdale, 2012). Eight, weekly, two-hour group sessions included teaching different mindfulness meditations, each followed by a discursive exploration of participants’ experiences (‘inquiry’). Every session would include a home practice review. The course included group support, psychoeducation, stories, videos and poetry. Specific references to the cognitive model were brought into Week 2 (cognitive theory and applications to tinnitus), Week 4 (cognitive model of tinnitus) and Week 6 (thoughts are not facts).

MBCT was modified to include more explicit reference to ‘mindfulness of sounds’ from Week 2, and explicit discussion of working with tinnitus in meditation. The cognitive model of tinnitus was used, based on McKenna et al. (2014), and videos were shown from patients who had completed MBCT-t. A workbook was given to all participants to support their practice and learning.

The eight-week course adhered to the following structure:

**Week 1:** Orientation to mindfulness, tinnitus and psychological well-being. Group building. Intention setting. Eating meditation (10 minutes) and body scan meditation (40 minutes). Home practice included 30 a daily body scan meditation plus informal mindful awareness of a routine activity and one meal.

**Week 2:** Body scan meditation (40 minutes). Psychoeducation (cognitive theory applied to tinnitus and how this relates to mindfulness). Sitting meditation (15 minutes) including awareness of sounds. Home practice included daily body scan and short daily sitting meditation, plus informal mindful awareness of a routine activity and noticing pleasant events.

**Week 3:** Mindful movement meditation (40 minutes) plus inquiry. Three-step breathing space (3 minutes). Sitting meditation, including awareness of sounds (15 minutes). Home practice included daily body scan or mindful movement (alternating) plus a ten-minute sitting meditation, three-step breathing space and noticing unpleasant events.

**Week 4:** Sitting meditation (40 minutes). Learning to use a three-step breathing space in response to difficulty. Introducing the Cognitive Model of Tinnitus. Video explaining selective attention and relationship to mindfulness. Home practice included daily mindful movement or sitting meditation (alternating) and three-step breathing space.

**Week 5**: Sitting meditation (with difficulty) (30 minutes). Group discussion on mindfulness, acceptance and exploring sounds. Video of MBCT- patient. Home practice involved daily sitting meditation and three-step breathing space.

**Week 6:** Sitting meditation (40 minutes) including awareness of thinking. Group discussion mindful responses to thinking (‘thoughts are not facts’) and related exercise. Home practice involved 30 – 40 minutes of formal meditation, three-step breathing space and noticing negative thoughts.

**Week 7:** Sitting meditation (40 minutes). Group discussion and exercise on ‘taking care of oneself’ and compassion. Loving kindness meditation (10 minutes). Home practice involved 30-40 minutes of formal meditation, breathing space and developing ‘early warning’ system.

**Week 8:** Body scan (40 minutes). Individual and group review of course. Developing a maintenance plan, including writing a letter to oneself. Home practice involved setting a regular formal meditation practice for the next month.

**One month follow up:** Review session including 30 minutes sitting meditation and plan how to continue with mindfulness meditation for the next five months.

**Six months follow up:** Review session including 30 minutes sitting meditation and review of past five months. Participants were informed of the interview study and invited to take part during this session.

1. **Summary of themes emerging from the IPA**

| **Supraordinate Themes** | **Subordinate Themes** | **Summary** |
| --- | --- | --- |
| **Relating to tinnitus in a new way** | Staying Present | Developing stable, flexible, open awareness and the ability to stay present with all experiences, including sound and tinnitus. |
|  | Equanimity (allowing and letting be) | Allowing tinnitus to be present, without fighting it or getting caught up in catastrophic thoughts about it. |
| **Holistic benefits** | Reduced distress | Reductions in negative thoughts, behaviours and emotions related to tinnitus and reductions in other types of distress such as low mood, stress, anxiety and interpersonal conflict. |
|  | Enhanced wellbeing | Improvements in wellbeing across many life-domains including sleep, energy, engagement with life and increases in ‘positive psychology’ experiences such as gratitude and joy. |
| **Connection, kindness and compassion** | With other people | The group was a community that acted as a vehicle for change and connection. Recognition of shared humanity with participants and teachers developed compassion, gratitude and appreciation one’s own and others’ blessings. The teachers’ professional and personal experience, and embodiment of attitudinal foundations of mindfulness fostered engagement, commitment, hope and learning. This extended out from the group and into the wider world. |
|  | With one’s self | Participants learnt to listen to themselves and meet their needs with kindness, compassion and permissiveness. |
| **Factors supporting engagement and change** | Factors that help and hinder the process | Inner and outer factors affected engagement. Sound meditation and applying of mindfulness to tinnitus was particularly helpful. There needed to be a balance between being open to trying new things, whilst also allowing and acknowledging scepticism. |
